# Self-Healing Flexible Conductive Film by Repairing Defects via Flowable Liquid Metal Droplets

**DOI:** 10.3390/mi10020113

**Published:** 2019-02-11

**Authors:** Ruiwen Niu, Mingliang Jin, Jieping Cao, Zhibin Yan, Jinwei Gao, Hao Wu, Guofu Zhou, Lingling Shui

**Affiliations:** 1 National Center for International Research on Green Optoelectronics, South China Normal University, Guangzhou 510006, China; niuruiwen@m.scnu.edu.cn (R.N.); jinml@scnu.edu.cn (M.J.); caojieping@m.scnu.edu.cn (J.C.); zhibin.yan@m.scnu.edu.cn (Z.Y.); gaojw@scnu.edu.cn (J.G.); haowu_cn@foxmail.com (H.W.); guofu.zhou@m.scnu.edu.cn (G.Z.); 2 Guangdong Provincial Key Laboratory of Optical Information Materials and Technology and Institute of Electronic Paper Displays, South China Academy of Advanced Optoelectronics, South China Normal University, Guangzhou 510006, China

**Keywords:** liquid metal, conductive film, self-healing, electrowetting

## Abstract

Self-healing flexible conductive films have been fabricated, evaluated, and applied. The film is composed of a fragile indium tin oxide (ITO) layer covered with sprayed liquid metal (LM) droplets. Self-healing of electrical conductivity is achieved via spontaneous capillary wicking of LM droplets into cracks/defects of the ITO film. The liquid metal adhering onto the ITO layer can also connect the ITO fragments during bending to keep the overall conductivity of the composite LM/ITO film stable. Stable and reversible electrowetting performance has been achieved with the composite LM/ITO as the conductive film, in either flat or curved states.

## 1. Introduction

Flexible and stretchable electronic devices have attracted enormous attention among emerging areas, including flexible displays [1], solar cells [2], radio-frequency identification (RFID) tags [3], flexible organic light-emitting diodes (OLED) [4], and portable and wearable electrical devices, including clothing or biomedical devices [5]. In these devices, flexible conductive film is one of the key components. Indium tin oxide (ITO) is commonly used as electrodes [6,7] due to its good conductivity and optical transparency. ITO on poly(ethylene terephthalate) (PET) substrate has been widely applied due to its high compliance and ruggedness [4]. Polymeric substrates like PET, polyethylene naphthalate (PEN), and polyimide (PI) are flexible; however, ITO film is inevitably stiff and brittle. In addition, poor adhesion of ITO to the polymer substrate makes the film easy to delaminate under compression or bending, which is also a major concern in manufacturing processes and during operation of flexible electronics. When subjected to tensile or fatigue loading, ITO-PET lamination would be susceptible to tensile micro-cracks, which in turn leads to the increase of sheet resistance of ITO film and the degradation of optical transmission [8,9,10]. In addition, ITO film has also been prepared on substrates using various complex and expensive deposition techniques, such as DC magnetron sputtering [11], chemical vapor deposition [12], spray pyrolysis [13], and pulsed laser deposition [12,14,15]. The adhesion and crystallization of the deposited material generally require a temperature above 300 °C; however, the thermos-stability of the organic substrate is poor, which brings certain difficulties to the film preparation. 

To meet the needs of flexible film development, numerous materials and strategies have been developed to replace or improve metal and ITO films in the past few years, including conductive materials (e.g., metal nanowire [2], carbon nanotube [16], graphene [17], and crack-nanonetwork [18]) and sandwich multilayer structures (e.g., ITO-metal-ITO [9,19]). However, one of the critical technical matters is the mechanical mismatch between common rigid conductive materials and soft supporting substrates, which leads to the formation of cracks or even delamination at interfaces of different materials upon an increased number of mechanical deformations. This has become one of the critical concerns for practical and long-term applications. 

As an alternative to these conventional approaches for flexible and stretchable electronics, the use of flowable liquid-phase conductors opens the path for all-soft, elastically deformable, shape-reconfigurable, and self-healing electronics [20,21]. Among conductive liquids, gallium-based liquid metal alloys have received particular interest because of their outstanding characteristics, such as relatively high electrical conductivity, low melting temperature, low toxicity, ultralow vapour pressure, and wide temperature range [20,21,22]. Physicochemical properties of these liquid metal alloys are summarized in Table S1, which also includes conventional conductive materials of pure gallium, mercury, water, and copper. Gallium-based liquid metal alloys can instantly form a self-limiting atomically thin layer of gallium oxide on a surface when exposed to air [23]. This thin oxide skin (0.7–3.0 nm) allows its good affinity to non-metallic surfaces without obviously influencing the thermal and electrical conductivities of bulk alloys [24,25]. Tremendous efforts have recently been devoted to fabricate stretchable and flexible electronics by using liquid metals as the conductive component [21,22,26], or as self-repairing materials for compensating traditional rigid conductive materials [20,27]. 

In this work, we take the advantages of the flowability and conductivity of gallium-based LM alloys to compensate for the disadvantages brought on by the mechanical defects and mismatch between rigid conductive films and soft polymeric substrates. An electrowetting on dielectric (EWOD) device, with a liquid-infused-membrane (LIM) as the flexible hydrophobic insulating layer, and a composite LM/ITO film, as the flexible conductive layer, have been fabricated and evaluated, showing excellent reversibility and low hysteresis of electrowetting performance. 

## 2. Materials and Methods

**Figure 1:**
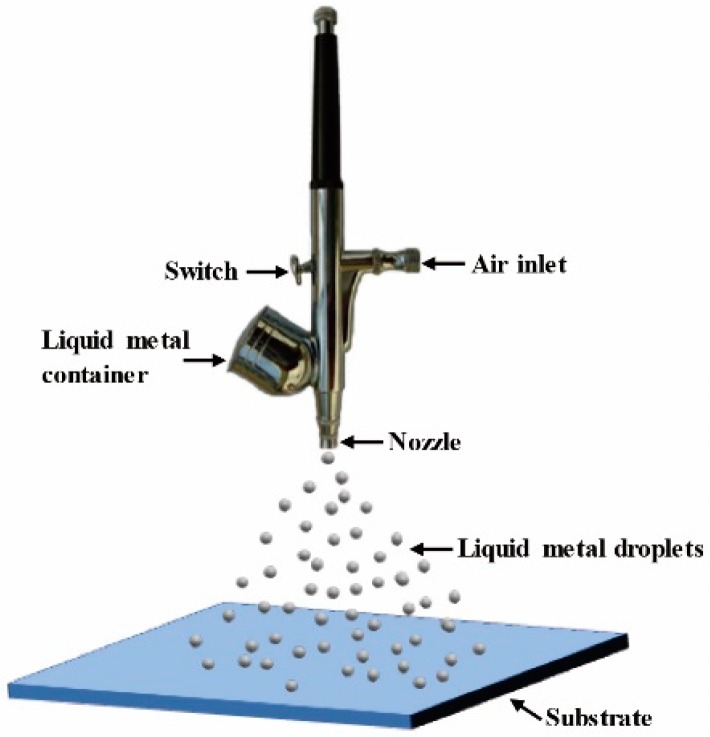
Schematic diagram of the preparation of liquid metal (LM) droplets and their deposition on a supporting substrate to produce a film.

The liquid metal of Galinstan, consisting of Ga, In, and Sn (Ga: In: Sn = 68.5: 21.5: 10 wt%), was purchased from Changsha Santech Materials Co., Ltd., Changsha, China. Its melting point is −19 °C, and its electrical conductivity is 3.46 × 10^6^ S/m at 20 °C. ITO/PET substrates (with ITO and PET thickness of 185 nm and 0.125 mm, respectively) were purchased from South China Xiang Cheng Technology Co., Ltd., Guangzhou, China. An airbrush was adopted for spraying the liquid metal (LM) droplets on substrates [25,28]. The substrate was carefully cleaned before depositing liquid metal, and the details can be found in the Supplementary Materials (SM). Figure 1 shows the schematic of the LM droplets preparation process. LM material was sprayed through a micro-nozzle fitted into the air pumping pipe. The nozzle diameter was 0.3 mm. The air produced by a pump entrained the LM fluid and atomized it into droplets. A switch beside the container controlled the amount of sprayed LM. The droplet size generated from a pneumatic nozzle is primarily a function of the nozzle diameter, gas pressure, and LM viscosity. Here, the switch was opened to the maximal degree to maintain constant flow ratio.

Sheet resistance of the conductive film was measured via two methods. One was the four-probe method [25] (ST2258C, Jingge Electronics Co., Ltd., Suzhou, China), which was used to measure the static sheet resistance of LM and LM/ITO films at different LM thickness (*λ*). The other was the two-probe technique [18] used to measure the dynamic resistance during bending tests. As shown in Figure S2, two copper strips are pasted onto the edge of the sample (3 cm × 3 cm). The bending fatigue specimen measured 30 mm in length and 30 mm in width, and the sheet resistance of the samples was measured using a Keithley 2400 Sourcemeter (Keithley, Cleveland, OH, USA). 

## 3. Results

### 3.1. Electrical Conductivity of the Films

**Figure 2:**
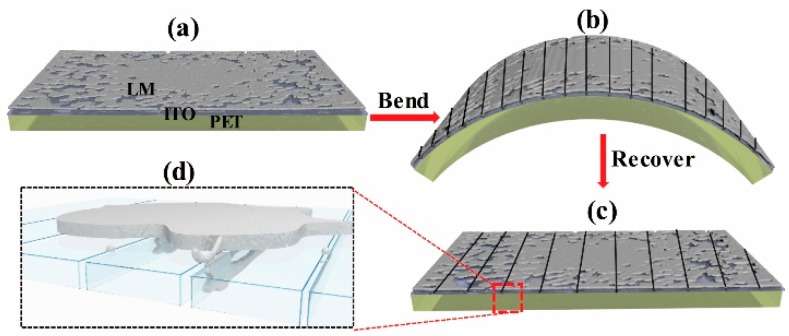
Schematic drawing of the structure and mechanism of the self-healing flexible conductive film. (**a**) Structure of the flexible film consisting of a poly(ethylene terephthalate) (PET) substrate (bottom), indium tin oxide (ITO) film (middle), and LM droplets (top). (**b**) Bending of flexible films creates multiple defects (crevices) on the ITO. (**c**) Repair of the defects via the conductive LM droplets, which can flow and fill in crevices via capillarity. (**d**) Magnified view of the LM filling in crevices.

Figure 2 shows the schematic of the construction and mechanism of the self-healing conductive film. LM droplets were prepared and deposited using the hand-held spraying pen (Figure 1), and the diameter of the LM droplets ranged from about 700 nm to 50 μm [25]. The composite LM/ITO film consisted of three layers, with the flexible PET as the supporting substrate, ITO film as the main conductive layer, and LM sprayed on top of ITO layer as the flowable conductive micro- and nano-droplets, as shown in Figure 2a. When the composite film was bent or stretched, the fragile ITO film was disrupted to form crevices (Figure 2b), destroying its electrical conductivity. However, according to the general principle of flowability, LM droplets flowed and filled in the crevices by capillary wicking (Figure 2c,d), thus repairing the ITO film which retained its conductivity. 

The amount of sprayed LM has been found to remarkably influence the electrical conductivity of the single-LM and composite LM/ITO films. The average thickness of the LM-layer was calculated by weighing tracing method, as calculated by Reference [29]: *λ* = *m*/(*ρS*), where *λ*, *m*, *S* are the thickness, quality, and area of the LM film, respectively, and *ρ* is the density of the LM. *λ* here just refers to an equivalent average thickness within the unit area by ignoring the film homogeneity. 

**Figure 3:**
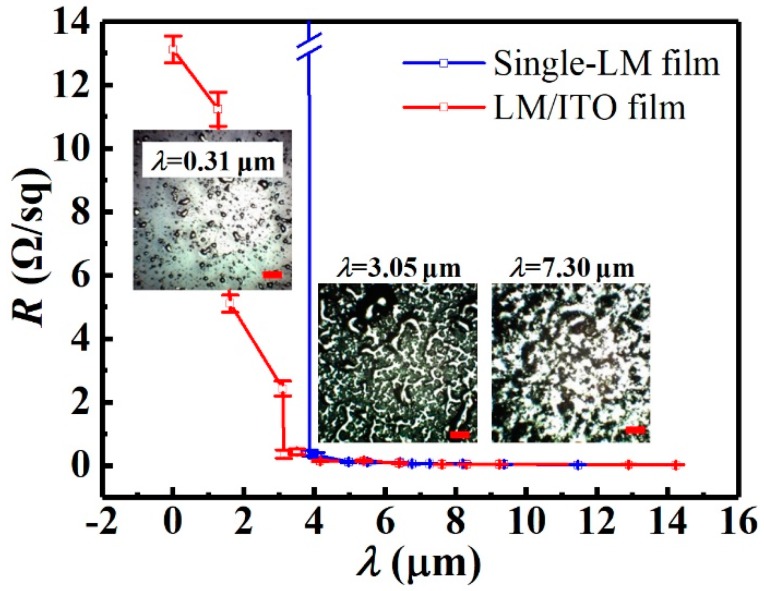
Sheet resistance (*R*) of the single-LM and LM/ITO films at different thicknesses (*λ*), with inset microscopic images of LM surfaces with *λ* of 0.31, 3.05, and 7.30 μm. Scale bars are 20 μm.

As shown in Figure 3, the formation of a LM film can be divided into three basic stages based on increased amounts of sprayed LM on a surface. LM droplets deposited onto a surface will form, stage by stage, (1) an initial intermittent island structure consisting of individual LM droplets, (2) a LM network connected by LM droplets, and (3) a continuous film composed of merged LM droplets. When LM was sprayed directly onto the PET substrate, a single LM film was either conductive or nonconductive depending on whether or not the LM droplets were connected to form a network (2) or continuous film (3).

It was evident that for the single LM film, when *λ* < 4.0 μm (on average), the sheet resistance (*R*) was too high to be measurable using our instruments (namely, nonconductive). For the composite LM/ITO, *R* was measurable, decreasing with the increase of *λ*, and reaching a plateau after a threshold value. In this work, since the electric conductivity of the LM material (3.4 × 10^6^ S/m) was higher than that of ITO (2.0 × 10^5^ S/m), LM led to more efficient conduction pathways than ITO. Therefore, for a composite LM/ITO film, *R* decreased from 13.12 (ITO film) to 0.36 Ω/sq when *λ* changed from 0 to 3.05 μm, and reached a constant value when the thickness was greater than 7 μm (about 0.04 Ω/sq). The constant value could be interpreted by the formation of a continuous conductive LM film (stage 2 or 3) on top of the ITO film. The effective sheet resistance of the composite film thus resulted from the in-parallel coupled LM and ITO layers reaching a stable value [30] (see Supplementary Materials). Therefore, the hybridization of LM with ITO produced a stable conductive film. The addition of liquid metal improved electrical conductivity, while it reduced the transmittance of the film due to the opacity of the liquid metal, as shown in Figure S1. However, with the excellent flexibility and high conductivity demonstrated by these films, people could have more options to choose among optical, electrical, and flexible properties for practical applications. 

### 3.2. Flexibility and Self-Healing of the Films

**Figure 4:**
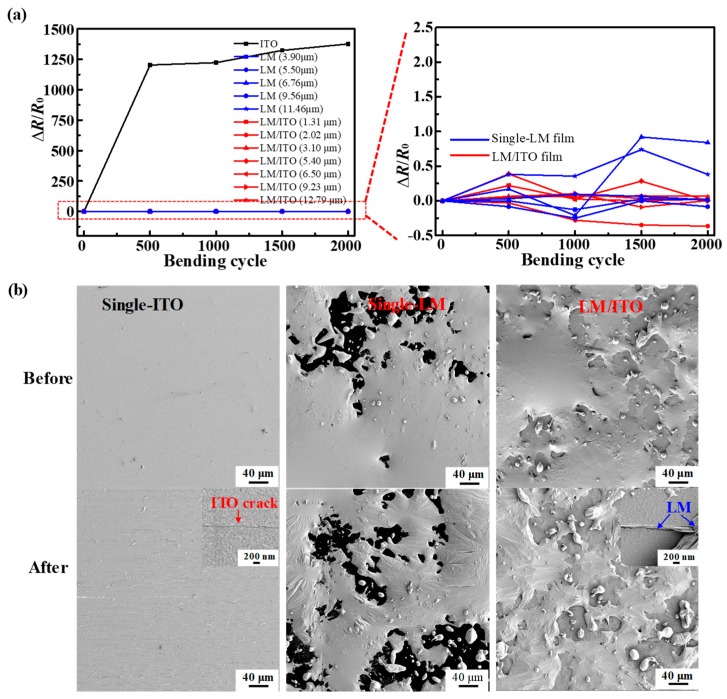
(**a**) Relative sheet resistance as a function of the bending cycles for the single-ITO, single-LM, and composite LM/ITO films. (**b**) SEM images of the single-ITO, single-LM, and composite LM/ITO film surfaces before and after the fatigue tests.

In order to comparatively evaluate the film flexibility, we examined and compared the fatigue performance of single-ITO, single-LM, and composite LM/ITO films on PET substrates, respectively, as demonstrated in Figure 4 and Figure S2. Figure S3a–c shows the sheet resistance varying with bending cycles from 0 to 2000. Figure 4a plots the relative changes of electric resistance (Δ*R*/*R*_0_, where *R*_0_ and *R* are the sheet resistance of a film before and after certain cycles of bending, and Δ*R* = *R*−*R*_0_) versus bending cycles for single-ITO, single-LM, and composite LM/ITO films. Δ*R*/*R*_0_ was over 1300 for ITO and below 1.0 for single-LM and LM/ITO during the 2000 cycles of fatigue tests. Obviously, the single-ITO film was destroyed after several bending cycles (Figure S3d); however, stable electrical conductivity was observed for the single-LM and LM/ITO films. Moreover, as shown in the magnified figure (Figure 4a), Δ*R*/*R*_0_ varied in the range of −0.3–1.0 and −0.1–0.4 for single-LM and LM/ITO films, respectively. The positive and negative values of the relative resistance correspond to increased and decreased resistance. This means that more reliable electrical conductivity was achieved with the composite LM/ITO films compared to the single-LM films. It should be noted that when *λ* was smaller than 3.0 μm, *R* was unmeasurable for the single-LM film. 

Figure 4b shows the SEM images of the single-ITO, single-LM, and composite LM/ITO films before and after the fatigue tests. Bare ITO surfaces were flat and smooth; however, many long and continuous cracks appeared after 2000 bending cycles, with their widths in the range of 10–150 nm. The single-LM film exhibited wrinkles on its surface after the bending test, which were caused by the LM droplets redistributing during the bending process, and the formation and displacement of the oxide surface film. Overall, the entire film remained continuous, and thus the LM film retained its circuit conductivity before and after bending when *λ* was over the threshold value. On the LM/ITO film, LM droplets were initially deposited on top of the ITO. After bending, various cracks appeared on the underlying ITO layer. However, LM was able to adhere to the ITO, flow to, and repair the conductive circuit by filling or joining adjacent cracks (Figures S4 and S5). The LM layer and ruptured ITO remained partially connected either in-parallel or in-series, as shown in Figure S6. Even at small *λ*, when LM did not cover the entire ITO surface, our experimental results demonstrated that this repairing mechanism was still preserved, keeping the overall conductivity of the film stable (Figure 4a and Figure S3). It is apparent that stable electrical conductivity can be achieved by compensating fragile ITO films with flowable LM droplets for long-term and reliable flexible electronic devices.

### 3.3. Electrowetting Performance on the Flexible Conductive Films

**Figure 5:**
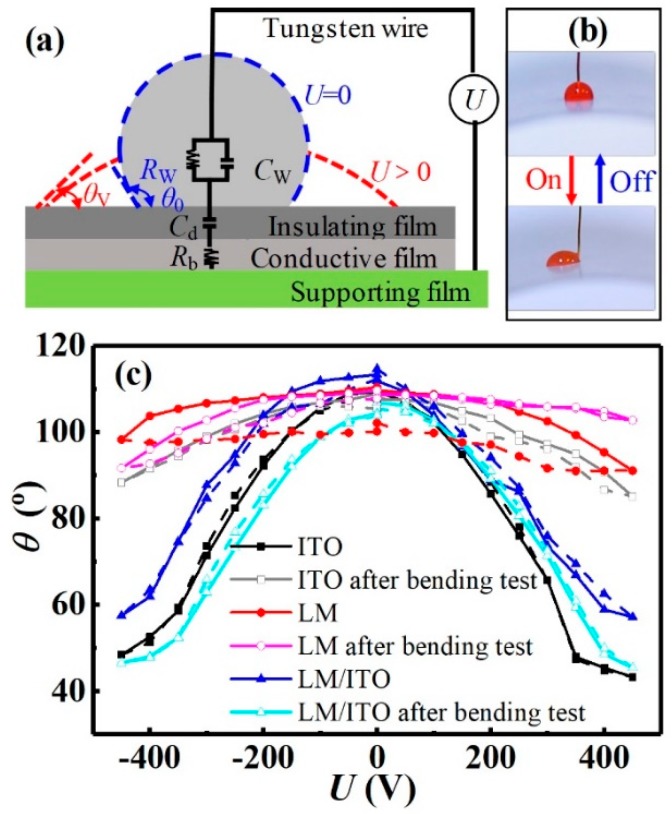
(**a**) Schematic of the electrowetting on dielectric (EWOD) device. The equivalent circuit diagram consists of a water droplet, a dielectric layer, and a conductive layer. (**b**) Optical images of a 2.0 μL water droplet on the top of a curved film, showing *θ* at *U* = 0 and 450 V. (**c**) EW performance of sessile drops on the liquid-infused-membranes (LIMs) with single-ITO, single-LM (*λ* = 3.11 μm), and composite LM/ITO (*λ* = 1.10 μm) films as the conductive layers.

To demonstrate the utility and reliability of these flexible conductive films, EWOD devices were fabricated with single-ITO, single-LM, or composite LM/ITO films as the flexible electrode layers on PET supporting substrates. A liquid-infused-membrane (LIM) made by infusing 3.0 μL silicon oil (5 cSt) into a 20 μm thick and 1.0 × 1.0 cm^2^ size PTFE membrane (Figure S7), was employed as the hydrophobic insulating layer [6]. Electrowetting-based digital droplet devices are one potential candidate for wearable biomedical and environmental monitoring devices for point of care (POC) and chemical oxygen demand (DOC) applications [31,32,33,34,35,36,37,38]. Most EWOD devices are based on rigid electrode and substrate materials, including gold, silver, copper, and ITO on glass or silicon substrates [37]. These materials are reliable and easily accessible in microelectronic areas; however, they are brittle and lack flexibility. Therefore, we used a flexible LIM directly stacked onto the as prepared LM/ITO surface to form a highly flexible EWOD device with a smooth surface without observable defects. A 2.0 μL DI water droplet was dripped on the LIM surface and driven by voltages applied via the tungsten wire connecting the droplet and the conductive film (Figure 5a). Figure 5b shows a sessile droplet contact angle change driven by an applied voltage of 0 and 450 V on a curved EWOD. Figure 5c plots the contact angle (*θ*) versus the applied voltage (*U*) on the LIMs with single-ITO, single-LM, and composite LM/ITO films as the conductive layers, and Figure S8 presents corresponding contact angles of sessile drops on different films driven by electric fields. *U* was varied between −450 to +450 V, at an increment of 50 V every 5 s. 

When using composite LM/ITO films, the EW performances were highly reversible and repeatable, with the *θ* difference of ~60° and unobvious *θ* hysteresis for all measurements at *λ* of 1.10–7.83 μm (Figure S9b). The single-ITO film showed stable and reversible EW performance before bending; however, it failed to show obvious EW performance after bending. On single-LM films, when *λ* was of ~2.0 μm, only a small *θ* change was observed, with a large *θ* discrepancy between increasing and decreasing *U* procedures (Figure S9a). This was mainly due to the limited contact area between the droplet and electrode caused by the discontinuous LM network, this being similar to that observed on the Ag-nanowire based conductive film (Figure S10). Although highly flexible conductive films could be obtained with conductive network structures, the electrical performance in local small areas failed. Therefore, the single-LM film with *λ* < 4 μm (network structure) is not suitable for applications requiring high contact area.

Electrowetting results were consistent with previously reported conductive films, considering the electrical properties and surface roughness [35,39]. Surface roughness was assumed to be constant using the same LIM. The equivalent circuit of EWOD was modeled as the capacitors of three layers in a series connection, as shown in Figure 5a. A direct current (DC) voltage was applied to charge the dielectric layer (with capacitance of *C*_d_) through the bottom electrode with resistance of *R*_b_. A water droplet with resistance of *R*_w_ and capacity of *C*_w_ lied between the bottom (conductive film) and the top electrode (tungsten wire). At the same *U*, the conductive film with higher resistance led to a larger voltage drop, which in turn reduced the voltage applied to the droplet and subsequently decreased the change in *θ* based on Lippmann’s equation [6,37]. Considering electrical conductivity, electrowetting performance, and costs, the critical LM amount with *λ* of 1.0–2.0 μm satisfied the requirements. Thus, the composite LM/ITO film was much preferred due to its high reliability. Table S2 summarizes the properties of LM film and LM/ITO film with different film thicknesses in terms of transmittance, electrical conductivity, flexibility, and electrowetting performance.

## 4. Conclusions

In this work, we have shown the fabrication, evaluation, and application of a flexible conductive film with electrical conductivity being self-healed after multiple and long-term bending fatigue tests. Self-healing of electrical conductivity is achieved via spontaneous capillary induced wicking of LM droplets into cracks/defects of ITO film to repair its electrical conductivity. Compared to single-ITO and single-LM films, the composite LM/ITO film shows stable and reliable electrical conductivity, with a sheet resistance of about 0.04 Ω/sq. A fully EWOD device has been demonstrated with highly reversible and stable contact angle change of about 60° (from 110° to 50°). Such a composite film with self-healing properties could be applied in flexible and sustainable electronic devices with increased fault-tolerance and circuit reliability, and extended service life.

## Supplementary Materials

The following are available online at http://www.mdpi.com/2072-666X/10/2/113/s1. **Figure S1:** Optical transmittance of (a) single LM and (b) composite LM/ITO films at various LM amounts. **Figure S2:** Photograph of the experimental bending fatigue system. **Figure S3:** Sheet resistance as a function of the bending cycles of (a) a single-ITO film, (b) a single-LM film, and (c) a composite LM/ITO film within 0–2000 cycles. (d) Sheet resistance versus bending cycles for single-ITO (black line) and composite LM/ITO (blue line) films at LM thickness *λ* of 1.31 μm within 0–50 cycles. **Figure S4**: SEM images of the surfaces after 2000 bending cycles of (a) LM films and (b) LM/ITO composite films. All scale bars are 20 μm. **Figure S5:** SEM images of the cracks in the LM/ITO composite film. **Figure S6**: Schematic of the LM and ITO connected (a) in-series and (b) in-parallel. **Figure S7:** SEM image of the PTFE membrane. **Figure S8:** Contact angles of sessile drops on multilayer EWOD films with the conductive layers of (a) single-LM and (b) composite LM/ITO films. (c) Comparison of contact angle changes on LIM surfaces with the single-ITO, single-LM, and composite LM/ITO films as the conductive layers, respectively, before and after the 2000-cycle bending tests. **Figure S9:** Contact angle changes with the applied voltage on the LIM surface with the single LM (a) and composite LM/ITO (b) films as the conductive layers, at various film thicknesses; **Figure S10**. Microscopic image of an Ag-nanowire based conductive film on a PET substrate. **Table S1**: Physicochemical properties of copper, mercury, gallium, EGaIn, Galinstan, and deionized water; **Table S2**: The transmittance, electrical conductivity, flexibility, and electrowetting performance of LM films and LM/ITO composite films with different film thicknesses.
