# Supplementary material for: Self-Healing Flexible Conductive Film by Repairing Defects via Flowable Liquid Metal Droplets
Source: Micromachines (Basel). 2019 Feb 11;10(2):113. doi: 10.3390/mi10020113 (PMC6412573; doi:10.3390/mi10020113)
Supplement: Supplementary file 1 [file micromachines-10-00113-s001.pdf]

Supplementary Information

Self-healing Flexible Conductive Film from Repairing Defects via Flowable Liquid Metal Droplets

Ruiwen Niu [1,2], Mingliang Jin [1,2], Jieping Cao [1,2], Zhibin Yan [1,2], Jinwei Gao [1,2], Hao Wu [1,2], Guofu Zhou [1,2] and Lingling Shui [1,2,*]

1. National Center for International Research on Green Optoelectronics, South China Normal University, Guangzhou 510006, China; niuruiwen@m.scnu.edu.cn (R.N.); jinml@scnu.edu.cn (M.J.); caojieping@m.scnu.edu.cn (J.C.); zhibin.yan@m.scnu.edu.cn (Z.Y.); gaojw@scnu.edu.cn (J.G); haowu_cn@foxmail.com (H.W.); guofu.zhou@m.scnu.edu.cn (G.Z.)
2. Guangdong Provincial Key Laboratory of Optical Information Materials and Technology and Institute of Electronic Paper Displays, South China Academy of Advanced Optoelectronics, South China Normal University, Guangzhou 510006, China

* Correspondence: Shuill@m.scnu.edu.cn; Tel.: +86-020-393-14813

Substrate Cleaning Process before Deposition Liquid Metal

The surface cleaning process before any deposition is necessary for the reproducible results without any spurious side effects such as the prior dirt particles on the surface of the substrate [1]**.** In order to reduce the measurement error, we performed the same cleaning process on the substrate each time the substrate was prepared. The PET and ITO/PET substrates arrived with thin polymer films that protect these substrates from scratches and contamination during transportation and storage. The protective polymer film must be removed prior to deposition of the liquid metal. The cleaning process can remove the surface dirt generated by the substrate production process. The cleaning process involves ultrasonic cleaning for 10 minutes, and then rinsing with ethyl alcohol (CH_3CH_2OH, analytical grade) followed by rinsing with ultrapure (UP) water and subsequently drying in N_2 (99.9%) gas flow.

Transmittance of LM Droplet Covered Films

In the present spraying method, small micro- and nano-droplets are deposited on substrates. Therefore, we speculate that such formed films may show certain optical properties, because the space among liquid metal droplets would provide some optical transmission, while the droplets are still connect with reserved electrical properties. Optical transmittance of these films were analyzed using an integrating spectrometer system (USB 2000, Ocean Optics, Orlando, Florida, USA). The transmittance data presented here are normalized to a reference substrate. Figure S1 depicts the optical transmittance of the films with various amount of LM. It is clear that the optical transmittance decreases when the LM amount increases for both single LM film and LM/ITO composite film, and reaches to the minimum transmittance of about zero when the surface is covered completely by continuous LM film.

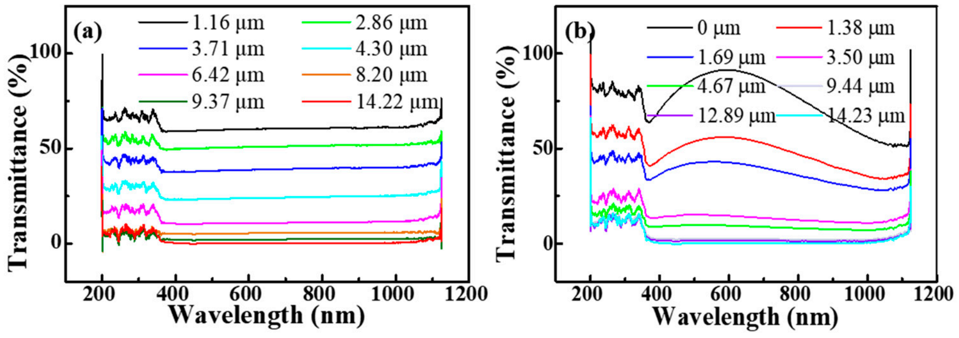

Figure S1. Optical transmittance of **(a)** single LM and **(b)** composite LM/ITO films at various LM amount.

Physicochemical Properties of Commonly Used Conductive Materials

Physicochemical properties of gallium-based liquid metal (LM) alloys are summarized in Table S1. Comparison to commonly applied conductive materials, including pure gallium, mercury, water and copper are included.

Table S1. Physicochemical properties of copper, mercury, gallium, EGaIn, galinstan and deionized water [2,3].

Property	Copper	Mercury	Gallium	EGaIn	Galinstan	DI water@ 25 ℃
Melting point (°C)	1085	−38.8	29.8	15.5	−19	0
Boiling point (°C)	2562	356	2205	2000	>1300	100
Density ($kg·m^{-3}$)	8960	13533	6093	6280	6440	998
Electrical conductivity ($S·m^{-1}$)	5.96×10^7	1.04×10^6	6.73×10^6	3.40×10^6	3.46×10^6	$<5.0 \times 10^{-4}$
Thermal conductivity ($W·m^{-1}·K^{-1}$)	-	8.5	29.3	26.6	16.5	0.6
Surface tension ($mN·m^{-1}$)	-	487	707	624	718	72
Vapor pressure (Pa)	-	1@42 °C	$\sim 10^{-35}$ @29.9 °C	N/A	$<1.33 \times 10^{-6}$ @500 °C	3169 @25 °C
Viscosity (Pa·s)	-	1.526×10^{-3}	1.37×10^{-3}	1.99×10^{-3}	2.4×10^{-3}	1×10^{-3}

Summary of the Properties of LM Film and LM/ITO Composite Film

As Table S2 shows, we summarize the performance of LM film and LM/ITO film with different film thicknesses in terms of transmittance, electrical conductivity, flexibility and electrowetting performance. It can be seen from Table S2 that when the thickness is small (λ < 4 μm), LM films and LM/ITO composite films have a certain transmittance, but the conductivity and flexibility of the LM films are poor in this thickness range, and the sheet resistance of the LM/ITO composite film is also large; when the thickness is large (λ > 4 μm), although the transmittance is greatly reduced, these two films have smaller sheet resistance, superior flexibility and better electrowetting properties. In practical applications, we can choose the appropriate film thickness based on different requirements for conductivity, light transmission, flexibility and electrowetting performance.

Table S2. The transmittance, electrical conductivity, flexibility and electrowetting performance of LM films and LM/ITO composite films with different film thickness.

	<4 μm		>4 μm	
**LM film**	Transmittance	30%–60%	Transmittance	< 30%
	Sheet resistance	∞	Sheet resistance	0.04–0.45 Ω/sq
	Flexibility	-	Flexibility	Good
	Electrowetting performance	Poor	Electrowetting performance	Good
**LM/ITO film**	Transmittance	10%–55%	Transmittance	< 10%
	Sheet resistance	0.36–13.12 Ω/sq	Sheet resistance	0.02–0.36 Ω/sq
	Flexibility	Good	Flexibility	Good

	Electrowetting performance	Good	Electrowetting performance	Good

Figure S2 shows the photograph of the two-probe system for bending fatigue test. This system can keep on bending the samples from plane to specific curvature radius for customized cycles setting. The bending velocity was set at 1 cycle/6 s, and the bending radius of fatigue test was set to be 3.50 mm.

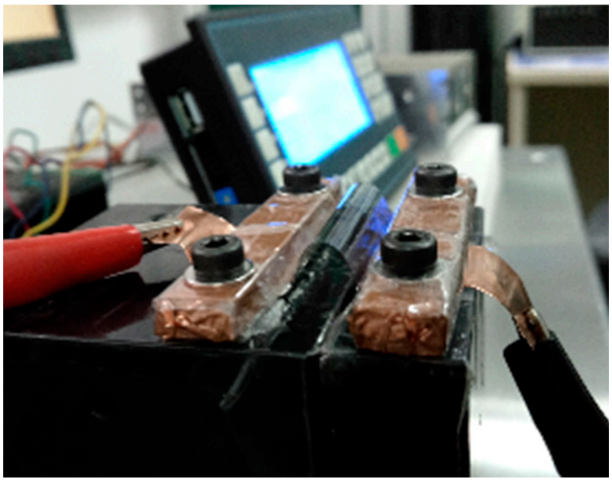

Figure S2. Photograph of the experimental bending fatigue system.

Figures S3a–c show the sheet resistance varying with bending cycles from 0 to 2000. With the increase of bending cycles, the sheet resistance of ITO film increases obviously to more than 104 Ω/sq; however, the sheet resistance of the composite LM/ITO film remains continuously at low values of less than 25 Ω/sq during the bending cycles. When the single ITO film was subject to bending fatigue loading, micro-cracks and defects were generated, and the measured sheet resistance exhibited degradation with increasing fatigue cycles as shown in Figure S3a. LM/ITO composite film is conductive even at small λ; therefore λ in the range of 1.31–11.46 µm were selected, showing typical sheet resistance of 0.04–30 Ω/sq (Figure S3c). As shown in Figure S3b, when $\lambda < 3.9$ µm the sheet resistance of LM film is too high to be measurable since LM droplets form island structure (1) without formation of network (2) or continuous connection (3). Therefore, the samples with λ of 3.90–11.45 µm were thus selected to investigate the electrical conductivity of single LM films with typical sheet resistance of < 5.0 Ω/sq. A local magnification of 0–50 cycles is shown in Figure S3d for more details. Unlike single ITO film, the selected LM and LM/ITO films did not show a continuous increase in resistance during the folding process when λ reaches a threshold value of about 4.0 µm. For the single LM film, the film continuity and density is not guaranteed since the substrate itself is nonconductive. For the composite LM/ITO film, conductivity can always be ensured since the ITO film itself is conductive, and the measurements have proven it as well. Moreover, more stable electrical conductivity has been achieved for the composite LM/ITO films than that of single LM films according to the smaller variation in the ratio of the sheet resistances after and before the bending tests.

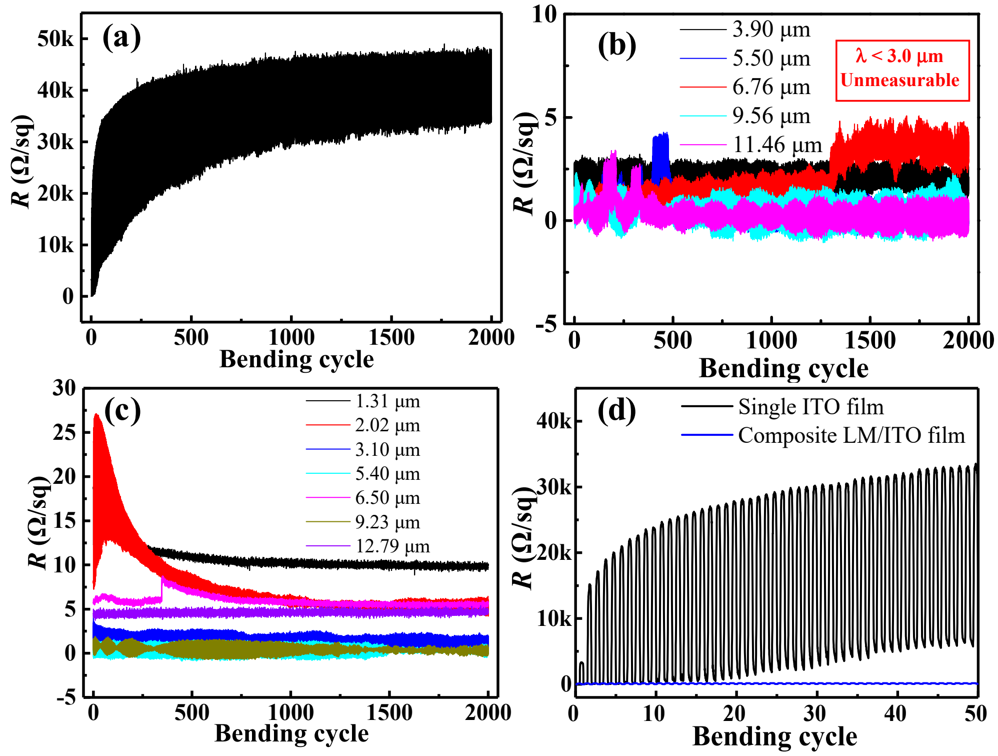

Figure S3. Sheet resistance as a function of the bending cycles of (**a**) a single-ITO film, (**b**) a single-LM film, and (**c**) a composite LM/ITO film within 0–2000 cycles; (**d**) Sheet resistance versus bending cycles for single-ITO (black line) and composite LM/ITO (blue line) films at LM thickness λ of 1.31 μm within 0–50 cycles.

Surface Morphology of the Constructed Conductive Films

The surface morphologies of the conductive films were examined using an optical microscope (Lissview, L1600, GuangZhou, China) and a scanning electron microscope (SEM) system (ZEISS Ultra 55, Carl Zeiss, Oberkochen, Germany). Figure S4 shows the surfaces of single LM films and composite LM/ITO films at different film thickness after 2000 bending cycles. It can be seen that after bending, there are a lot of wrinkles on the surface of the LM film, and as for the LM/ITO composite film, cracks appear in the underlying ITO layer, and the liquid metal in the upper layer is wrinkled. Figure S5 shows the SEM images of the ITO cracks in the folded LM/ITO composite film, it can be seen that the liquid metal appears in the crack or cover the crack.

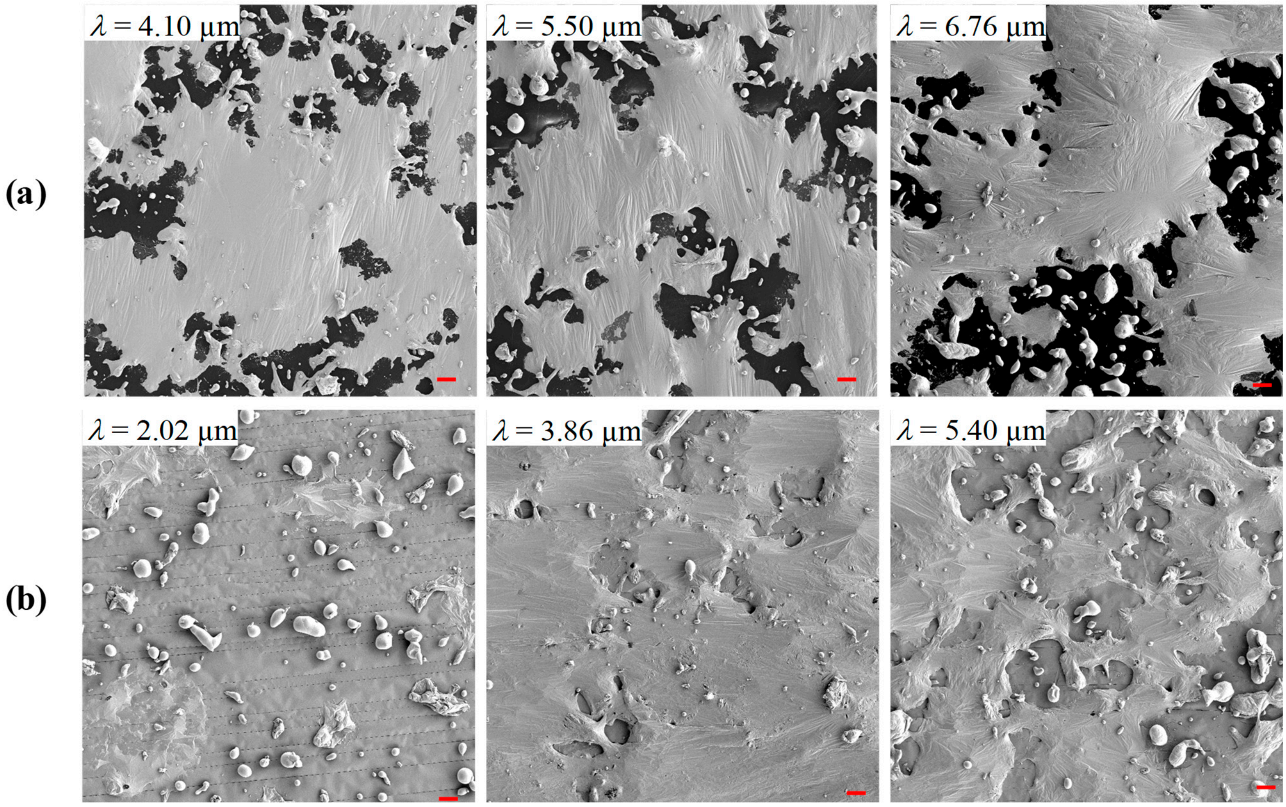

Figure S4. SEM images of the surfaces after 2000 bending cycles of (**a**) LM films and (**b**) LM/ITO composite films. All the scale bars are 20 μm.

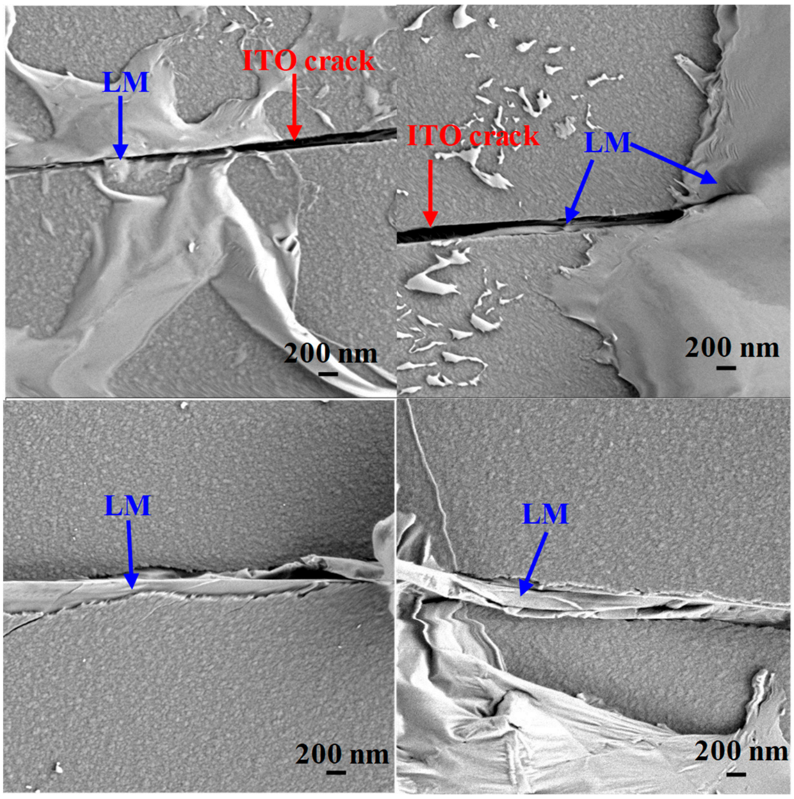

Figure S5. SEM images of the cracks in the LM/ITO composite film.

Connection Modes of LM and ITO in the LM/ITO Composite Films

In this work, the LM materials are assumed to be pure without contamination, and the thickness of LM film formed locally is uniform. When LM flows into cracks/crevices of the ITO film, LM and ITO are connected in-series. When LM is covered on the ITO layer as a continuous film, LM and ITO are connected in-parallel. The schematic drawing is presented in Figure S6.

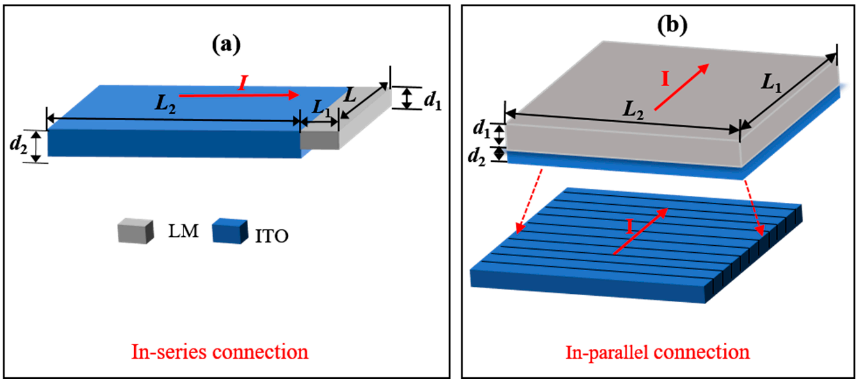

Figure S6. Schematic of the LM and ITO connected **(a)** in-series and **(b)** in-parallel.

According to the two basic electric resistance Equations (1) and (2) as follows:

$$R=\frac{\rho \cdot L}{S} \tag{1}$$

$$R=\frac{\rho \cdot L_1}{d \cdot L_2} \tag{2}$$

ρ, S and L are the resistivity, cross-sectional area and length of the conductive materials, respectively. L_1 and L_2 are the film length along and perpendicular to the current flow direction, and d is the film thickness. The total resistance of the films connected in-series can be calculated using the Equation (3):

$$R_{\mathrm{total}} = \frac{L_1}{L} \times \frac{(\rho_1\text{-}R_{\mathrm{sq}} d_1)}{d_1} + \frac{R_{\mathrm{sq}} L_c}{L} \tag{3}$$

The total resistance of the films connected in-parallel can be calculated using the Equation (4):

$$R_{\mathrm{total}} = \frac{\rho_1 R_{\mathrm{sq}} l_1}{d_1 l_2 R_{\mathrm{sq}} + l_2 \rho_1} \ . \tag{4}$$

ρ_1 is the resistivity of the Resistor 1 (LM, here), L_1 and L_2 are the length of LM and ITO films along the current direction, respectively, d_1 is the thickness of LM, R_{sq} is the sheet resistance of ITO film, L_c is the sum of L_1 and L_2.

As seen from Equation (4), the total resistance of in-parallel connection is constant since the electric conductivity of LM material (3.4×10^6 S/m) is much higher than that of the ITO film (2×10^5 S/m). On the other hand, it means that the more in-series connection, the smaller the total resistance will be. Thus, when the LM film thickness is high enough to form a continuous film, or the bending induced film redistribution to connect discontinuous LM droplets of segments to a continuous film, the sheet resistance becomes stable.

Characterization of the Electrowetting Performance on the Flexible Films

The mechanism of self-healing conductivity of LM/ITO films on PET substrates has been achieved via LM droplets filling into an ITO crack by capillary wicking and connect adjacent cracks with in-series or in-parallel. To evaluate the reliability and applicability of the constructed flexible films, the multilayer EWOD device was built by stacking a flexible liquid-infused-film (LIF) on top of a conductive film. The combination of two fluidic

materials of liquid metal and silicone oil in the LM/ITO conductive and LIF hydrophobic insulating layer, respectively, show excellent flexibility and compatibility. The EWOD devices show smooth surfaces without gas bubbles. Contact angles were measured using an OCA 15 Pro contact-angle system (Dataphysics, Germany). Voltages were applied via two tungsten wires with one end connected to a power source (PSW, China) and the other end connected to the droplet or conductive film. Figure S7 is a scanning electron microscope (SEM) image of the PTFE membrane for constructing the LIF, representing the porous structure for liquid filling possibility. The average pore size and thickness of the PTFE membrane are ~200 nm and ~20 μm, respectively.

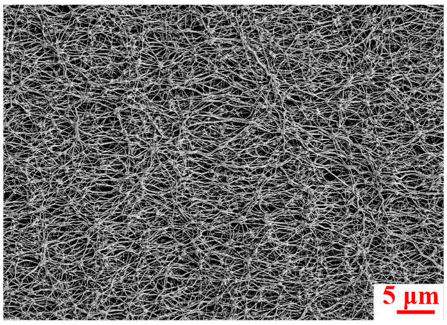

Figure S7. SEM image of the PTFE membrane.

Figure S8 presents corresponding contact angles of sessile drops on different films driven by electric fields. Figure S8a shows that, with the increase of LM film thickness of the single-LM films, the contact angle difference increases from 27 to 60° driven at 450 V. Figure S8b demonstrate the stable electrowetting performance which is not obviously affected by the LM film thickness for the composite LM/ITO films. The comparison shown in Figure S8c is consistent with previously presented results in this work.

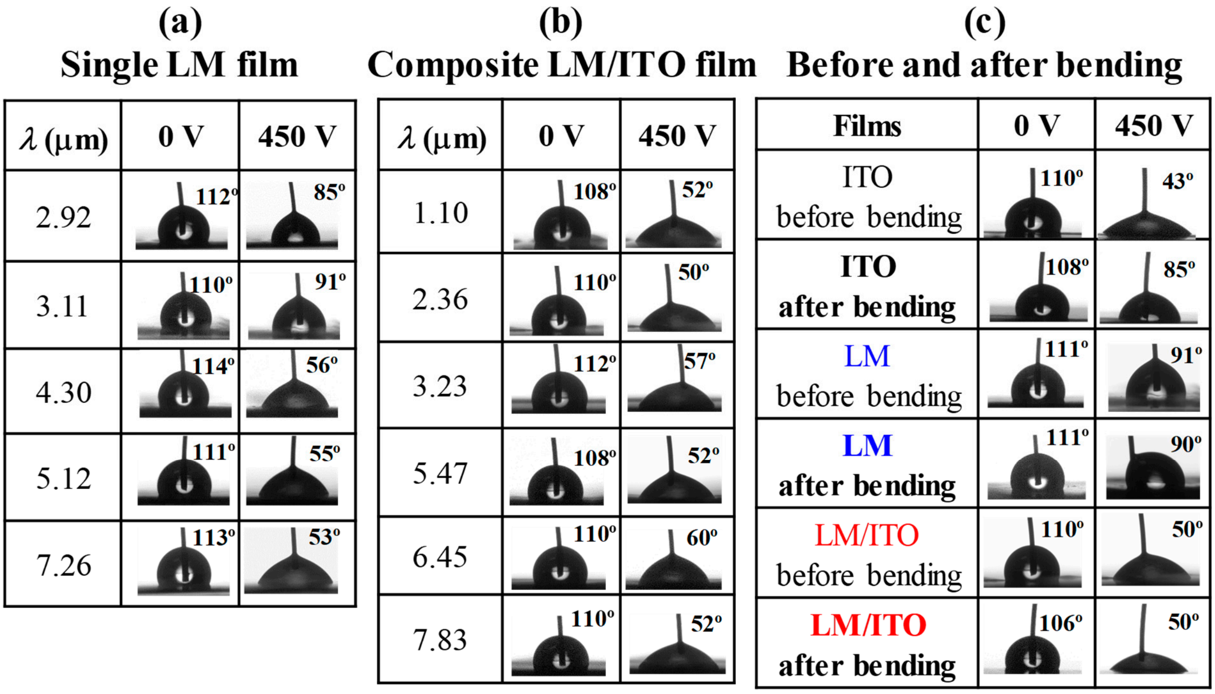

Figure S8. Contact angles of sessile drops on multilayer EWOD films with the conductive layers of **(a)** single-LM and **(b)** composite LM/ITO films; **(c)** Comparison of contact angle changes on LIF film with the single-ITO, single-LM and composite LM/ITO films as the conductive layers, respectively, before and after the 2000-cycle bending tests.

On single-LM films, the electrowetting performance was only preserved when the LM thickness is >4 μm, as shown in Figure S9a. In Figure S9b**,** reversibly electrowetting performance was observed on all LM/ITO films as conductive layers.

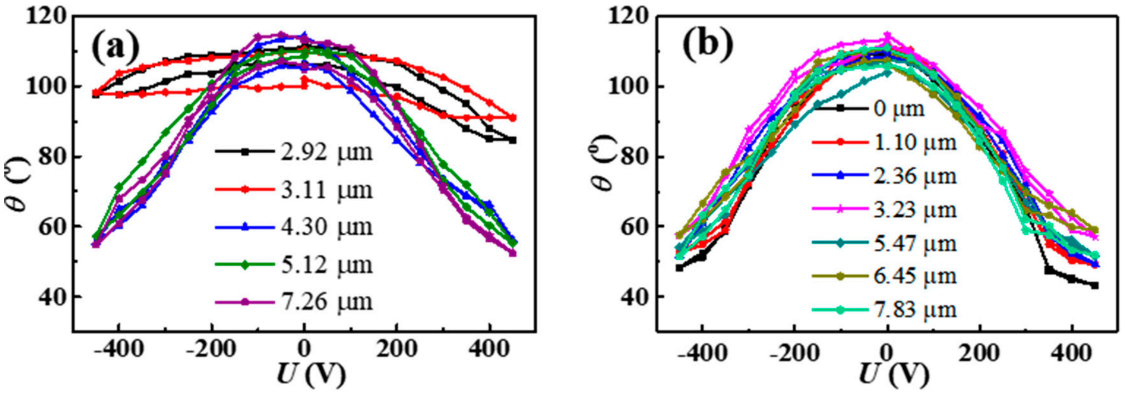

Figure S9. Contact angle changes with the applied voltage on the LIF surface with the single LM (**a**) and composite LM/ITO (**b**) films as the conductive layers, at various film thickness.

Figure S10 shows a microscopic image of an Ag-nanowire based conductive film. Although the whole film is conductive, most area is still empty according to the network structure. Thus, only the contact area of the water droplet and the conductive wires works to drive the droplet. As a result, only slight contact angle change of about 10° (decreases from 110 to 101° before saturation) could be achieved using such a network conductive film to drive a sessile droplet via electrowetting mechanism.

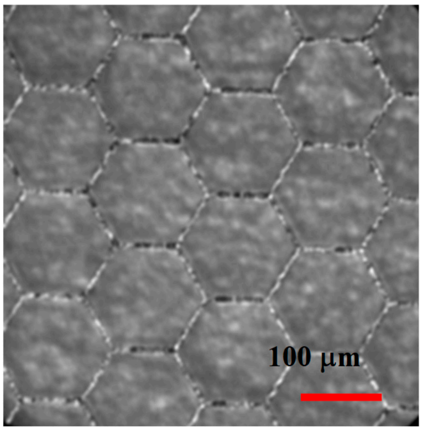

Figure S10. Microscopic image of an Ag-nanowire based conductive film on a PET substrate.

References

1. Malviya, K.D.; Dotan, H.; Yoon, K.R.; Kim, I.-D.; Rothschild, A. Rigorous substrate cleaning process for reproducible thin film hematite (α-Fe_2O_3) photoanodes. *J. Mater. Research* **2016**, *31*, 1565–1573.
2. Wu, Z.; Hjort, K.; Jeong, S.H. Microfluidic stretchable radio-frequency devices. *P. IEEE* **2015**, *103*, 1211–1225.
3. Khoshmanesh, K.; Tang, S.-Y.; Zhu, J.Y.; Schaefer, S.; Mitchell, A.; Kalantar-zadeh, K.; Dickey, M.D. Liquid metal enabled microfluidics. *Lab Chip* **2017**, *17*, 974–993.

© 2019 by the authors. Submitted for possible open access publication under the terms and conditions of the Creative Commons Attribution (CC BY) license (http://creativecommons.org/licenses/by/4.0/).
